# Investigating the relationship between sleep and migraine in a global sample: a Bayesian cross-sectional approach

**DOI:** 10.1186/s10194-023-01638-6

**Published:** 2023-09-08

**Authors:** Emily C. Stanyer, Jack Brookes, Jia Rong Pang, Alexandre Urani, Philip R. Holland, Jan Hoffmann

**Affiliations:** 1 Wolfson Centre for Age-Related Diseases, Institute for Psychiatry, Psychology, and Neuroscience, King’s College London, London, UK; 2 Current address: Sleep and Circadian Neuroscience Institute, Nuffield Department of Clinical Neurosciences, University of Oxford, Oxford, United Kingdom; 3 Independent Researcher, London, UK; 4 Healint Pte Ltd, Singapore, Singapore; 5 NIHR-Wellcome Trust King’s Clinical Research Facility/SLaM Biomedical Research Centre, King’s College Hospital, London, UK

**Keywords:** Migraine, Headache, Sleep, Pain, Bayesian, Modelling, Sleep deprivation

## Abstract

**Background:**

There is a bidirectional link between sleep and migraine, however causality is difficult to determine. This study aimed to investigate this relationship using data collected from a smartphone application.

**Methods:**

Self-reported data from 11,166 global users (aged 18–81 years, mean: 41.21, standard deviation: 11.49) were collected from the Migraine Buddy application (Healint Pte. Ltd.). Measures included: start and end times of sleep and migraine attacks, and pain intensity. Bayesian regression models were used to predict occurrence of a migraine attack the next day based on users’ deviations from average sleep, number of sleep interruptions, and hours slept the night before in those reporting ≥ 8 and < 25 migraine attacks on average per month. Conversely, we modelled whether attack occurrence and pain intensity predicted hours slept that night.

**Results:**

There were 724 users (129 males, 412 females, 183 unknown, mean age = 41.88 years, *SD* = 11.63), with a mean monthly attack frequency of 9.94. More sleep interruptions (95% Highest Density Interval (95%HDI [0.11 – 0.21]) and deviation from a user’s mean sleep (95%HDI [0.04 – 0.08]) were significant predictors of a next day attack. Total hours slept was not a significant predictor (95%HDI [-0.04 – 0.04]). Pain intensity, but not attack occurrence was a positive predictor of hours slept.

**Conclusions:**

Sleep fragmentation and deviation from typical sleep are the main drivers of the relationship between sleep and migraine. Having a migraine attack does not predict sleep duration, yet the pain associated with it does. This study highlights sleep as crucial in migraine management.

**Supplementary Information:**

The online version contains supplementary material available at 10.1186/s10194-023-01638-6.

## Background

There is an established relationship between sleep and migraine [1, 2], and sleep and pain [3]. For example, migraine attacks are often preceded by premonitory symptoms, including fatigue [4]. Paradoxically, sleep disruption, both too little or too much is reported to trigger attacks in approximately 50% of patients [5] and represents a risk factor for chronification [6]. Conversely, patients report sleep as a strategy for attack resolution [7]. This is complicated further as many treatments affect the sleep cycle [8] and affective disorders involving sleep disturbance are three times more likely in people with migraine than the general population [9]. In a meta-analysis, we reported poorer subjective sleep quality and reduced rapid-eye-movement (REM) sleep in migraine patients compared to healthy controls [10]. However, what remains unanswered is whether poor sleep increases the likelihood of migraine attacks or whether having an attack causes disrupted sleep. This is important as migraine is a disabling disorder impacting over one billion people globally [11]. Whilst progress has been made with CGRP-based treatments [12], a significant proportion of patients remain “non-responders” to such therapies [13], thus understanding this relationship could lead to identification of further novel therapies and treatment strategies.

Previous research demonstrated that hours slept on the previous night was predictive of pain [14] and migraine [15] the next day. Conversely, other studies have reported that sleep fragmentation, but not reduced hours slept, increases pain [16], and is associated with higher odds of a migraine attack the next day [17]. Alternatively, jet-lag, shift work [18, 19] and changes to the surrounding environment are key migraine triggers [20], Thus, reduced total sleep time (TST) may not precipitate attacks, yet deviation from usual sleep might. Supporting this, shift work is associated with higher likelihood of developing chronic pain [21]. To our knowledge, no studies have investigated the impact of attacks on sleep. Although, studies have demonstrated that pain results in poor subsequent sleep including reduced TST, frequent awakenings, and sleep quality [22–24]. Therefore, it is likely that pain during migraine attacks reduces TST.

Whilst studies have attempted to elucidate this relationship, many have small sample sizes, focus on one population at one time point, include biased samples (e.g. patients reporting to clinics for co-morbid sleep complaints), and provide conflicting findings. However, it is important to understand directionality, to explore mechanisms and identify novel treatments.

The current study aimed to reconcile this disparity and investigate this within a global sample using self-reported measures collected from a smartphone application. This analysis differs from the previous literature as it avoids issues with retrospective reporting, as users report migraine attacks when they happen, and sleep measures are automatically detected by the application, and this is the first study to investigate the impact of migraine on sleep. We hypothesize that more sleep interruptions [17], and shorter sleep duration the night before, will predict the occurrence of a migraine attack the next day [15]. Secondly, we hypothesize that deviation from average monthly sleep duration the night before, will predict the occurrence of an attack the next day [19]. Lastly, we hypothesize that experiencing an attack and associated pain intensity will both predict a reduction in hours slept compared to average sleep on the evening the attack begins [25, 26].

## Methods

### Design and inclusion criteria

This study followed the STROBE reporting guidelines (see Supplementary Table 1). This was a retrospective cross-sectional study on self-reported data gathered from the Migraine Buddy mobile application (Healint Pte. Ltd., https://healint.com). Data were collected between 30^th^ June 2021 and 31^st^ December 2021. Only application users which were active for at least six months and reported an activity in each month during the collection period were included. Six months' worth of data was chosen as it avoids issues with seasonal triggers and circannual periodicity [27]. Users were required to have signed up to the application before the 7^th^ January 2021 and had to have 25 or more days of user-confirmed sleep records in each month during the period. For data analysis purposes, we excluded users with < 8 and > 25 attacks per month on average. There were no restrictions on the geographical location, age, or gender of the participants and these were optional for the participant to report. No ethical review board was required due to there being no user-identifiable information collected or stored. When users signed up to the application they agreed to the terms and conditions of the Healint Pte. Ltd. policy of data collection and disclosure.

### Data transformation and pre-processing

The following self-reported variables were gathered by the application: demographics (if reported: age, gender, country), start and end times of each sleep episode, and start and end times of each reported migraine attack, whether an attack was thought to be triggered by menstruation by the participant (yes/no), the highest pain intensity for each migraine attack (visual analogue scale 0–10), and the symptoms experienced with each attack. It is important to note that users could report the attack as and when it started, or retrospectively. Thus, associated pain measurements could also be retrospective, and these could be updated during an attack or after. For the purposes of demographic reporting, users were categorised by age as follows: young adults < 40 years, and older adults ≥ 40 years. Participants could choose from a range of default symptoms for each attack or alternatively use a free-text response to enter a non-default symptom.

To account for the different time zones in the dataset, times (sleep and migraine attack start and end times) were converted from Coordinated Universal Time (UTC) to the user’s local time zone. The mean number of attacks per month over the six months for each participant was computed from the start and end times of attacks. For the purposes of demographic reporting, the percentage of attacks which were reported to be triggered by menstruation was calculated for each individual.

### Measurement of sleep

The measurement of sleep by the application is based upon when a user picks up their mobile phone during the night, thus if they do not pick up their phone, they are assumed to be asleep. The user confirms their estimated hours slept detected by the application the following morning, and only confirmed sleep records were included in the dataset. This measurement of sleep is not the gold standard, however, smartphone applications are thought to be valid for detecting TST and binary sleep and wake parameters [28], and in the current study we were interested in overall prediction of sleep duration and fragmentation.

The number of hours slept was calculated from the start and end times and number of unique sleep episode identifiers. Based on the start time of the sleep, it was assigned to a night, and defined as the same night if a sleep started before 08:00 AM. This aims to avoid two sleep episodes (e.g. one beginning at 11:59 and another at 00:01) being classified as a different night’s sleep. Based on this information, if multiple sleep episodes were recorded on one night this was counted as a sleep interruption. To generate the number of interruptions variable we used the number of separate sleep episodes per night minus one. The time in bed variable was calculated as the difference between the start time of the first sleep episode of the night and the end time of the final sleep event for that night. Wake time was calculated as the time in bed minus the number of hours slept. Sleep efficiency was calculated as time asleep divided by time in bed multiplied by 100 based on convention. To calculate deviations from monthly mean hours slept, for each user we computed a Z-score of their total hours slept and took the absolute value of the Z-score of an individual’s deviation, and for each night calculated how many standard deviations away from the mean the sleep duration was.

Whilst users could not directly report naps in the application, they were unlikely to have been counted in the sleep duration measurements. The app uses automatic sleep detection; however, users set parameters for when they want their automatic detection to activate and deactivate e.g. 22:00–08:00. Thus, the app would not automatically detect naps during the day, as well as sleep that was earlier or later than these times. However, users must confirm their sleep the next morning thus they could adjust if their sleep was outside these pre-set times.

### Measurement of migraine

A similar approach was taken to classify migraine attacks. Each migraine episode and its duration was classified based on the self-reported start and end time. Users who reported on average more than 25 migraine episodes per month were excluded (*n* = 61). If users had daily attacks, it would be difficult to establish attack onset and fit a predictive model. Similarly, for the Bayesian analysis, we removed users reporting < 8 attacks per month due to the difficulties fitting a predictive model with infrequent attacks. We removed any individual migraine attacks which were greater than 96 h, as typical attacks last between 4 and 72 h [29] and these were likely to be data entry errors. As migraine attacks typically span multiple days, we calculated the duration of migraine experienced on each day as well as the date that the attack started. For example, if it started at 20:00 on one day but ended at 16:00 the next day attack duration would be classified as four hours on day one, and 16 h on day two. For all variables the mean and standard deviation were computed.

### Bayesian modelling

We used Bayesian estimation techniques to infer distributions of possible parameter values in each model. We chose a Bayesian approach rather than traditional frequentist statistics as this avoids issues surrounding the interpretation and arbitrary nature of *p*-values and statistical significance [30, 31]. Bayesian linear regression allows quantification of evidence in favour of, or against, a substantive model compared with a baseline model [32].

To establish whether sleep variables could predict occurrence of a migraine attack on a given day, we created a generalized linear model with a constant plus three predictor variables: deviation from an individual’s mean sleep duration (*Z*-score), number of sleep interruptions, and total hours slept, and whether an attack occurred or not (yes/no) as the binary dependent variable. Conversely, we created a linear model supposing that total hours slept on a given night could be predicted from a constant plus two predictor variables: occurrence of an attack (0 or 1) and pain intensity of an attack (0 – 10).

We used Bayesian inference to estimate the posterior distribution of every parameter value, which quantifies the relationship between that predictor variable and the outcome variable. In both models, parameter values were estimated per participant, and assumed to come from a normal distribution, with the mean and standard deviation of the distribution also being estimated.

We implemented these analyses using the *BRMS* package in *R. BRMS* uses Stan and implements a Hamiltonian Markov Chain Monte Carlo algorithm. We estimated the posterior distribution *P*(Parameters | Data) using the No-U-Turn algorithm implemented in *BRMS.* For the first model with attack occurrence as the outcome variable, Bernouli was used as the family function, and for the second where hours slept was the outcome variable, the default Gaussian was used. Four separate chains of 4,000 samples were taken from the posterior distribution, the first 1,000 samples were discarded as warmup samples. We verified the chains did not diverge using trace-plots after sampling, and $\widehat{R}$ values were close to 1.000 in both models for all parameters. Parameters’ priors were all set to be uninformative normal distributions with mean = 0 and standard deviation = 100.

We computed 95% highest density intervals (HDIs) on population mean parameters, plotted posterior distributions as a density plot, and used the positioning in relation to 0 to assess the likelihood of a variable being a significant predictor. Posterior distributions are a probability distribution which represent our updated beliefs (uncertainty or certainty) about the parameter/s after having seen the data, combined with what we knew before the data were collected (the prior belief). In this case, as we used uninformative priors, the posterior distribution is driven entirely by the data. Posterior means of the β-value estimates for each model were computed.

## Results

### Demographics

**Fig. 1:**
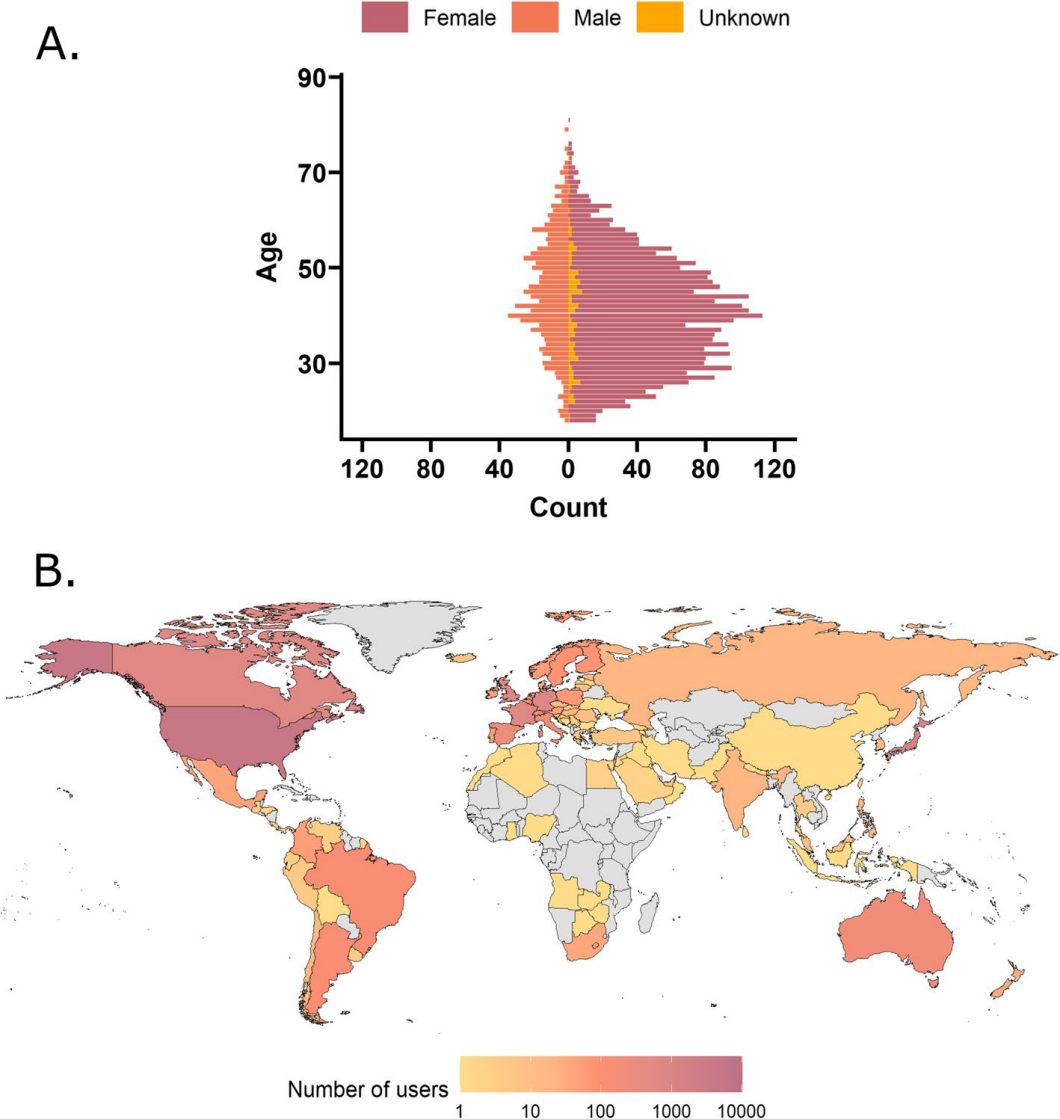
Demographics of users in the Migraine Buddy dataset. **A** The population of users mirrors that seen in epidemiological studies with a much higher prevalence of migraine in females, with a peak around 30–40 years of age. **B** Choropleth map showing the distribution and number of users across the world, with most users based in the USA, Canada, Japan, Germany, UK and France. Countries in grey are those without any users in the current dataset

There were 11,166 app users in the dataset. We removed any users which reported their age as < 18 years as sleep architecture can change with age [33] and we were interested in studying the relationship between sleep and migraine in adults. As the app does not require reporting of age or gender, of the 11,086 users, 7239 did not report their age, leaving age data for 3847 users. The mean age of the 3847 users which reported it was 41.21 years (range = 18–81 years old, SD = 11.50; Fig. 1). Importantly, users which did not report their age or demographics were not excluded from the analysis, as these variables were not included in any analyses but simply used for demographic reporting. There may have been users under 18 years of age which did not disclose their age, therefore it is possible that some users under 18 were included in the analysis. When categorizing adults as younger (< 40 years of age) and older (≥ 40 years), there were 1729 younger adults and 2118 older adults.

Of the 11,086 users, 5911 self-reported as female (53.32%), 1276 as male (11.51%), and 3899 did not report their gender (35.17%). This demographic data mirrors that seen in epidemiological studies[34] with a much higher prevalence of migraine in females, and a peak in prevalence around 30–40 years of age.

**Fig. 2:**
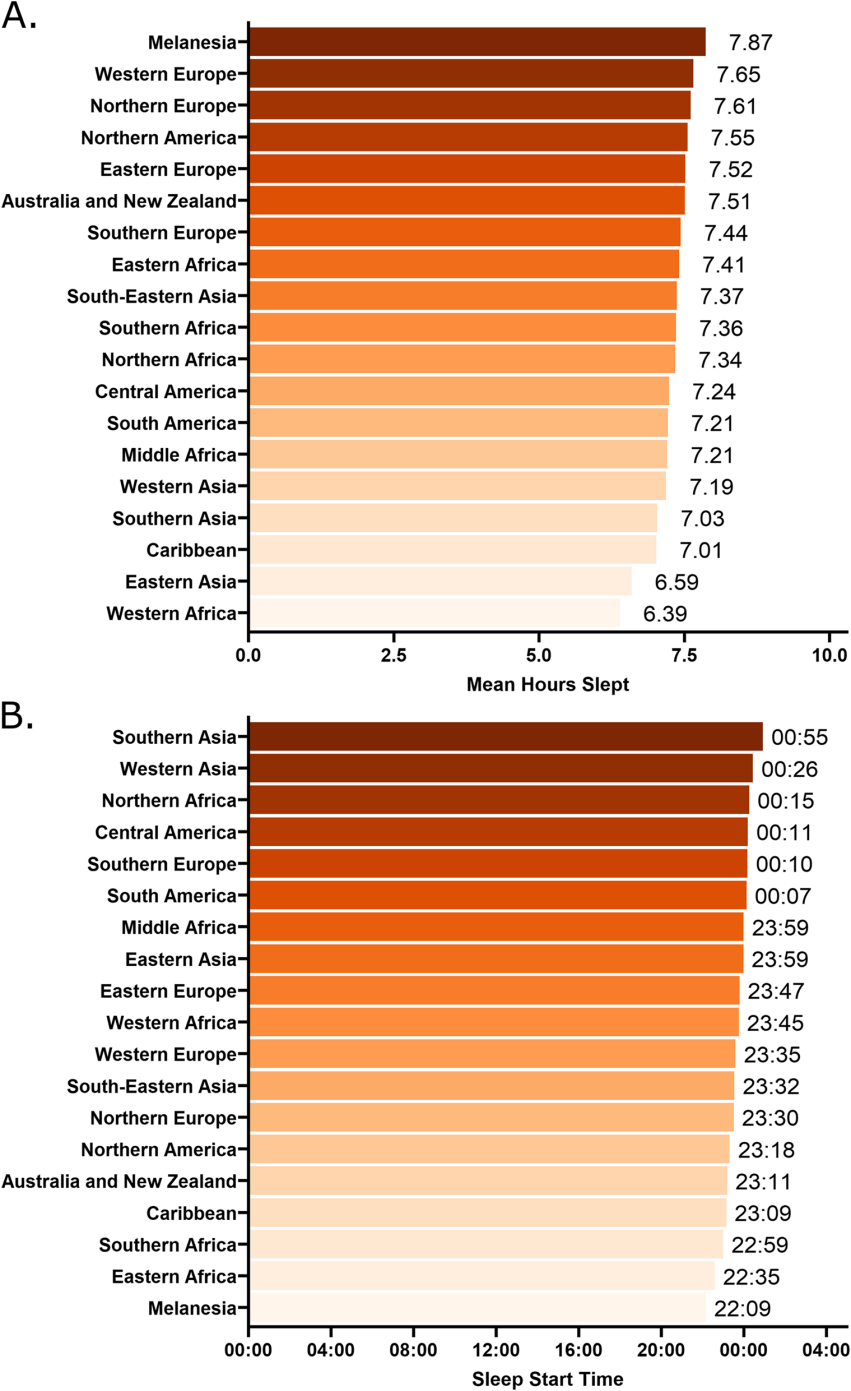
Sleep characteristics according to geographical region for the overall sample. **A** Mean hours slept according to region, with the shortest sleep duration (6.39 h) in Western Africa and the longest duration in Melanesia (7.87 h). **B** Mean sleep start time according to region. The latest sleep onset time was in Southern Asia (00:55) and the earliest was in Melanesia (22:09)

**Fig. 3:**
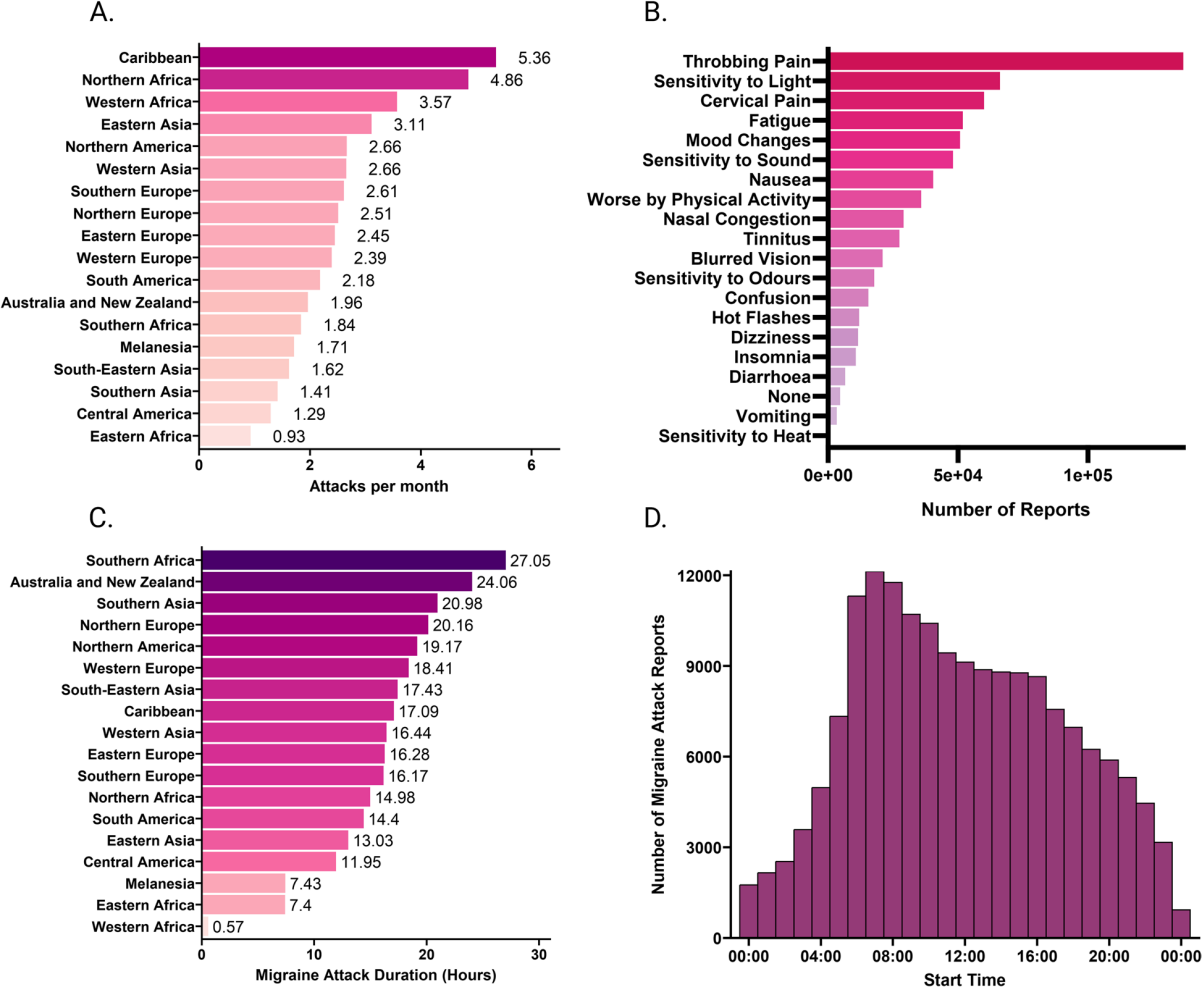
Migraine characteristics for the overall sample. **A** Mean number of attacks per month according to geographical region across the whole sample. **B** Self-reported symptoms associated with all reported attacks across the entire sample. **C** Migraine attack duration in hours according to region. **D** Histogram of the start time of all reported migraine attacks, not specific to individuals

Users were reportedly based in 99 different countries (Fig. 1) with most of the app users residing in the United States of America (39.34%), Japan (10.54%), Germany (6.68%), United Kingdom (6.43%), and France (6.29%) at the time at which they signed up to the app. Sleep (Fig. 2) and migraine variables (Fig. 3) are plotted according to geographical region.

### Sleep and migraine attack characteristics

The descriptive statistics for the sleep and migraine measures according to age group and gender are shown in Tables 1 and 2 respectively.

**Table 1:** Descriptive statistics for the migraine variables for the overall sample

	**Overall sample** **(*n* = 11,086)**	**Older** **Adults** **(*n* = 2118)**	**Young** **Adults** **(*n* = 1729)**	**Age unknown** **(*n* = 7239)**	**Female** **(*n* = 5911)**	**Male** **(*n* = 1276)**	**Gender unknown** **(*n* = 3899)**
Pain intensity (VAS)	5.26 (1.64)	5.34 (1.63)	5.50 (1.55)	5.19 (1.65)	5.42 (1.59)	4.90 (1.77)	5.14 (1.64)
Attack duration (hrs)	18.15 (14.86)	19.46 (15.65)	17.91 (13.75)	17.70 (14.71)	19.16 (15.22)	14.69 (13.07)	17.72 (14.65)
Number of attacks over 6-month period	19.77 (24.17)	21.72 (24.94)	18.14 (21.87)	19.32 (24.22)	20.62 (24.44)	23.41 (27.71)	17.27 (22.21)
Menstruation as trigger (%)	0.23 (0.29)	0.18 (0.28)	0.31 (0.30)	0.24 (0.29)	0.27 (0.30)	0.0 (0.02)	0.25 (0.29)
Attacks per month	2.82 (3.45)	3.10 (3.56)	2.59 (3.12)	2.76 (3.46)	2.95 (3.49)	3.34 (3.96)	2.47 (3.17)
Migraine start (hrs)	11.95 (3.31)	11.46 (3.14)	12.96 (3.18)	11.96 (3.35)	5.42 (1.59)	4.89 (1.77)	5.14 (1.64)
Migraine end (hrs)	30.12 (15.10)	30.91 (15.88)	31.07 (14.04)	29.66 (14.95)	19.18 (15.25)	14.77 (13.10)	17.73 (14.66)

Data are represented as mean (standard deviation)

*Abbreviations*: *hrs* hours, *VAS* Visual analogue scale. Migraine start time is from midnight

**Table 2:** Descriptive statistics for the sleep variables for the overall sample

	**Overall sample** **(*n* = 11,086)**	**Older** **Adults** **(*n* = 2118)**	**Young** **Adults** **(*n* = 1729)**	**Age unknown** **(*n* = 7239)**	**Female** **(*n* = 5911)**	**Male** **(*n* = 1276)**	**Gender unknown** **(*n* = 3899)**
TST	7.45 (1.00)	7.46 (1.04)	7.53 (0.95)	7.43 (1.00)	7.55 (1.00)	7.36 (0.96)	7.34 (1.01)
Sleep Start Time (hrs)	23.55 (1.34)	23.40 (1.42)	23.75 (1.41)	23.55 (1.29)	23.50 (1.38)	23.62 (1.35)	23.60 (1.27)
Sleep End Time (hrs)	31.21 (1.24)	31.10 (1.27)	31.54 (1.23)	31.17 (1.22)	31.28 (1.25)	31.15 (1.31)	31.13 (1.19)
Time in Bed (hrs)	7.66 (1.22)	7.71 (1.28)	7.79 (1.27)	7.62 (1.18)	7.78 (1.25)	7.53 (1.10)	7.54 (1.18)
Time Awake (hrs)	0.21 (0.69)	0.25 (0.76)	0.26 (0.78)	0.19 (0.64)	0.23 (0.75)	0.17 (0.51)	0.19 (0.63)
Sleep Efficiency (%)	99.00 (0.03)	98.00 (0.04)	98.00 (0.04)	99.00 (0.03)	99.00 (0.04)	99.00 (0.03)	099.00 (0.03)
Sleep Interruptions	0.12 (0.18)	0.13 (0.20)	0.13 (0.18)	0.12 (0.18)	0.12 (0.19)	0.10 (0.17)	0.13 (0.17)

Data are represented as mean (standard deviation)

*Abbreviations*: *hrs* hours, *TST* Total sleep time

### Bayesian modelling

**Fig. 4:**
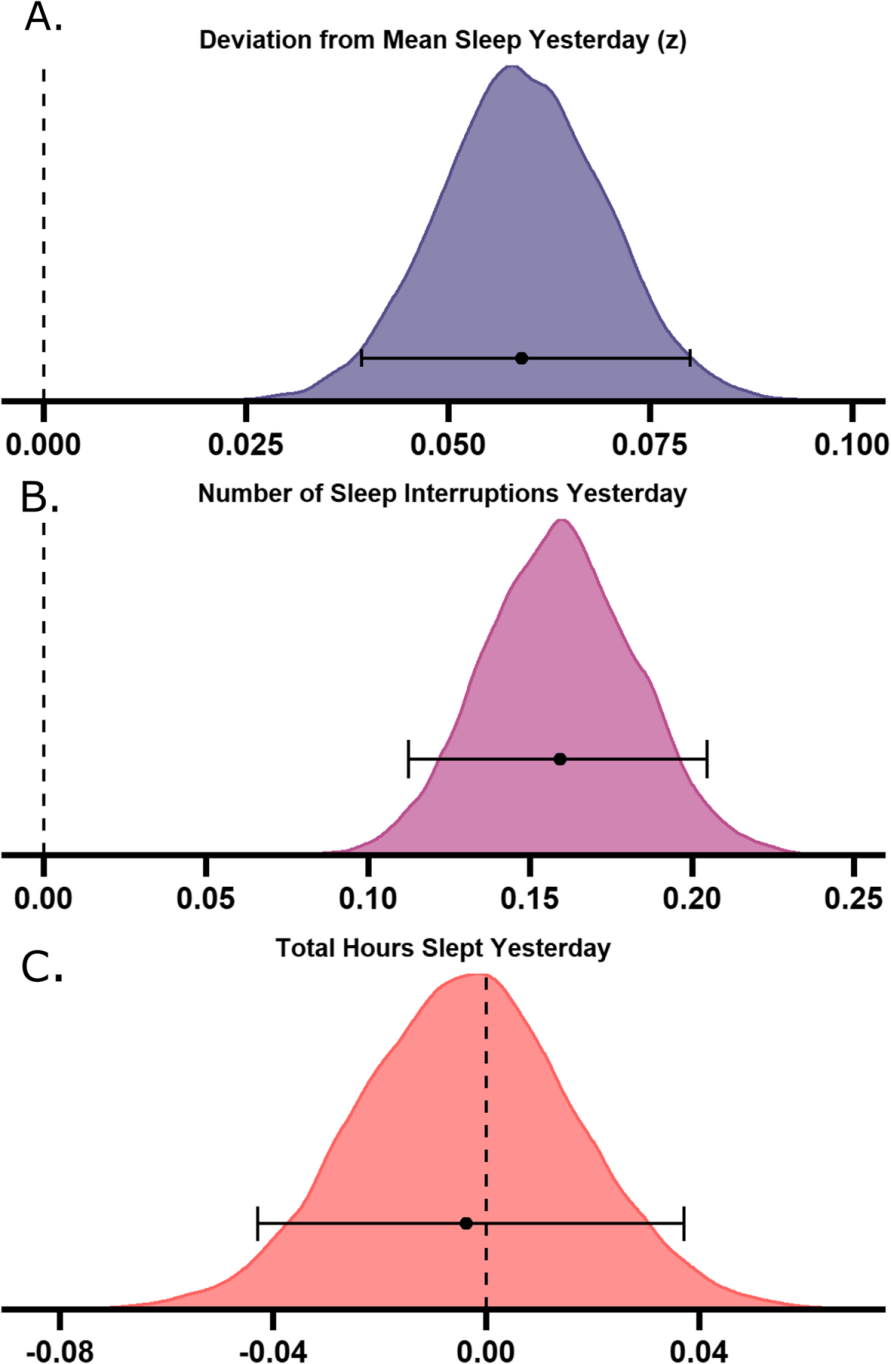
Bayesian posterior distributions for sleep variables predicting migraine attacks. Larger deviations from typical sleep (**A** *M* = 0.06, 95% highest density interval (HDI) [0.04 – 0.08]) and a greater number of sleep interruptions (**B** *M* = 0.16, 95% HDI [0.11 –0.21]) predict occurrence of a next day migraine attack, but total hours slept does not (**C** *M* = -0.00, 95% HDI [-0.04 – 0.04]). Density plots show posterior distributions for the population means of the three tested sleep-related predictor variables on whether a migraine attack occurred or not. The x-axis represents b-value estimates of the parameter. Error bars indicate the 95% highest density interval, with the central point indicating the mean of the posterior distribution

**Fig. 5:**
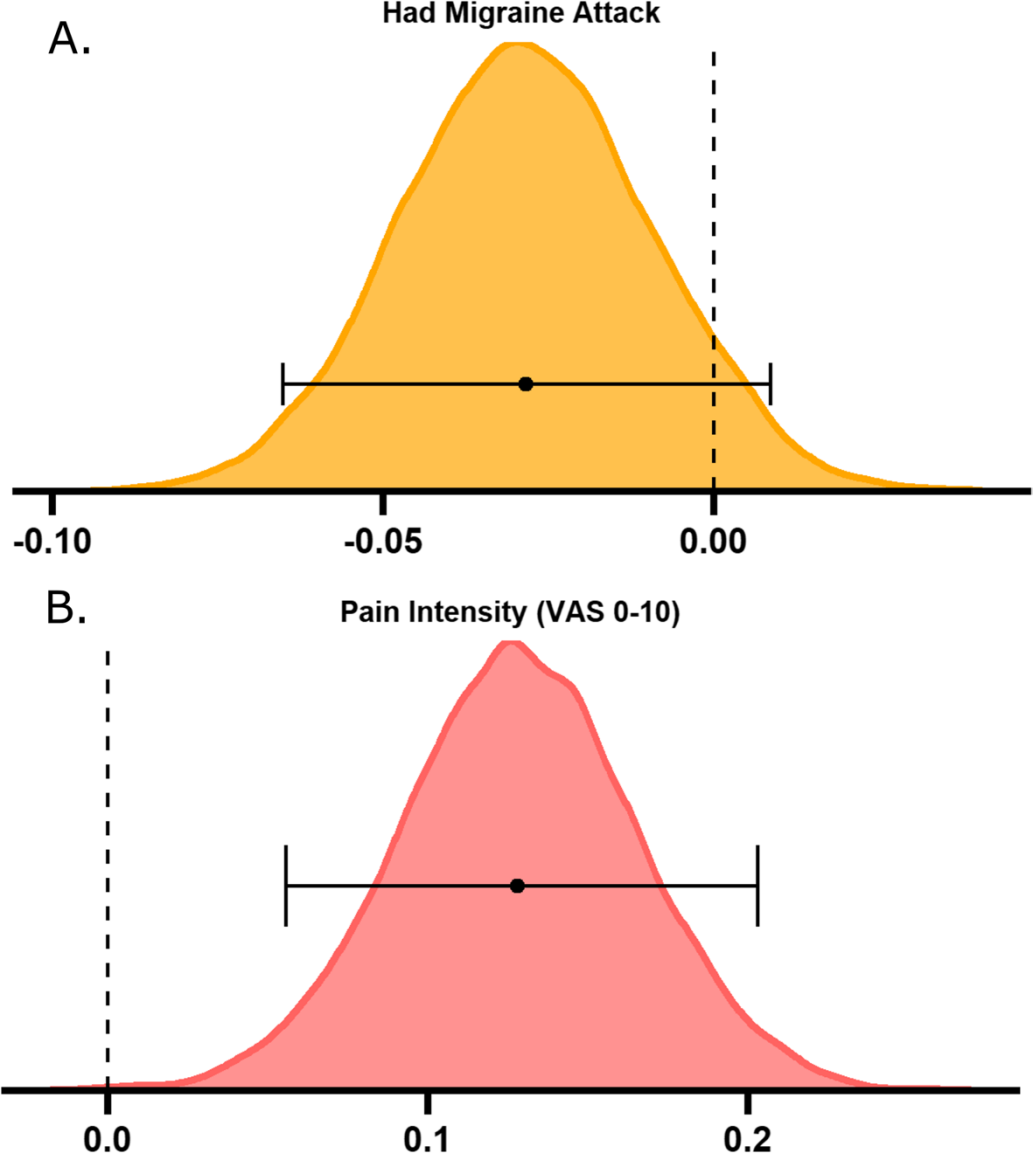
Bayesian posterior distributions for migraine variables predicting sleep duration. Having an attack did not predict alterations to sleep (**A** *M* = -0.03, 95% highest density interval (HDI) [-0.07—0.01]), but greater pain intensity (**B** *M* = 0.13, 95% HDI [0.06—0.20]) results in more hours slept the same night. Density plots show posterior distributions for the two tested models of the population means of migraine-related predictor variables on total hours slept. Plots represent the posterior distribution, density estimate. The x-axis represents b-value estimates of the parameter. Error bars indicate the 95% highest density interval. VAS = visual analogue scale

Ten thousand three hundred sixty-two users were excluded based on the migraine frequency requirements (those with ≤ 8 and ≥ 25 attacks per month). Seven hundred ninety-four users were left if only the ≤ 8 criteria were used, thus only 70 users were excluded with ≥ 25 attacks on average per month, suggesting that the main reason for exclusion was too few attacks per month. Thus in the Bayesian modelling there were 724 users (129 males, 412 females, 183 gender unknown) with an average age of 41.88 years (SD = 11.63) and mean monthly attack occurrence of 9.94. Posterior distributions show the population mean parameter fits across the two models (see Figs. 4 and 5). These distributions tell us the estimate of the possible values of the parameters given the data.

### Sleep parameters predicting migraine

We sought to explore whether sleep variables could predict a next day migraine attack. Deviation from mean sleep duration in hours (*M* = 0.06, 95% highest density interval (HDI) [0.04 – 0.08]) and number of sleep interruptions yesterday (*M* = 0.16, 95% HDI [0.11 –0.21]) were positive predictors of next day migraine attack occurrence, as assessed by the positioning in relation to 0. This means that for every deviation from mean sleep, there is a 6.1% increase in odds of having an attack, and for every sleep interruption, there is 17.4% increased odds of an attack occurring. Total hours slept yesterday was not a positive or negative predictor of attack occurrence (*M* = -0.00, 95% HDI [-0.04 – 0.04]).

### Migraine parameters predicting sleep

Next, we aimed to investigate the opposite relationship – whether reporting a migraine attack and the self-reported pain associated with that attack could predict alterations in sleep duration. Having a migraine attack was not a predictor of hours slept that evening (*M* = -0.03, 95% HDI [-0.07—0.01]), whereas the pain intensity reported during an attack was a positive predictor (*M* = 0.13, 95% HDI [0.06—0.20]), indicating that for every increase of 1 on the VAS for pain intensity, hours slept increased by 0.13 h (~ 8 min).

## Discussion

This analysis aimed to investigate the relationship between sleep and migraine in a global sample using measures collected from a smartphone application. Using Bayesian regression models, we demonstrated that fragmented sleep, and deviation from typical sleep predict the occurrence of a migraine attack the next day but sleep duration does not. Thus, we were able to partially support our hypothesis. On the other hand, reporting a more painful migraine attack is predictive of increased sleep duration that same evening, but having a migraine attack is not. This contrasts with our hypothesis as we hypothesized that both having an attack and a more painful attack would predict reduced sleep duration. Thus, we were able to disentangle this bidirectional relationship, in that poor sleep increases the risk of next-day migraine occurrence, but having a migraine attack is not sufficient to disrupt sleep. However, the pain intensity associated with attacks alters sleep.

Firstly, we demonstrated that sleep interruptions but not hours slept was predictive of migraine. This aligns with previous work whereby sleep fragmentation but not hours slept was predictive of migraine [16, 17], as well as with literature in which sleep fragmentation predicts, or leads to pain [35, 36]. Surprisingly, we found that sleep duration was not a predictor of migraine attack occurrence. Previous studies demonstrated that short sleep duration predicts pain [14] and migraine occurrence [15]. Moreover, sleeping too much is a reported trigger for migraine [37], yet our findings do not support this.

Unexpectedly, we found that more painful attacks resulted in longer sleep duration. Previous literature has shown that pain results in reduced total TST [22–24]. This also aligns with a prospective questionnaire study in pediatric migraine which found that headache intensity was a significant predictor of delayed or disturbed sleep [38]. Although we did not include children in the present investigation, this feature may be conserved from children to adults. This finding raises the question of potential mechanisms. During sleep the brain is thought to clear out waste metabolites via glymphatic clearance [39]. Theories suggest a role of perturbed energy metabolism or mitochondrial dysfunction in headache pathophysiology [40]. Interestingly, cortical spreading depression (CSD) – the proposed neurophysiological correlate of migraine aura, results in poor glymphatic flow [41], and increased non-rapid eye movement sleep [42, 43]. Whilst migraine with and without aura may be subserved by different mechanisms, it is possible that the increased sleep duration observed herein following attack initiation results from attempts to restore metabolic imbalance and facilitate glymphatic clearance of metabolites. Future studies are required to elucidate this.

Deviation from typical sleep duration the night before predicted a next day migraine attack. Whilst no studies have not explored this directly, this aligns with reports of shift-work and jet-lag triggering migraine [19]. Thus, the timing and regulation of sleep rather than the absolute duration of sleep, may be important for migraine initiation. Indeed, evidence has uncovered potential circadian mechanisms underlying attack initiation [44].

### Strengths & limitations

Despite significant exclusions and reduced sample size from the initial cohort based on frequency requirements, our final sample size remained large in comparison to existing studies. Further strengths are that there was little potential for forgetting as patients reported the start and end times of migraine attacks in real time. Users in the final analysis were based in 38 different countries, across a six-month period. This is in comparison to previous studies which often focus on one region and time point suggesting that the results are generalizable to a wide range of countries and cultures.

However, there are some limitations to consider with this study. Most notably, we used estimates of sleep from a smart phone application which determines whether someone is asleep based on whether they pick up their mobile phone. This means we could fail to capture interruptions where the individual did not go on their mobile phone, and subtler changes to sleep architecture. However, users have to approve the estimate of their sleep episode the next morning suggesting that it provides an accurate subjective reflection of their sleep, and smartphone applications have been shown to give valid estimates of TST [28]. Nonetheless, these results show that despite the cause of the sleep disruption (intrinsic or extrinsic), it can impact migraine likelihood. Moreover, even with this coarse-grained measure we were able to see that changes in sleep are a significant predictor of migraine attacks, suggesting the utility of datasets collected from smartphone applications for investigating neurological conditions. Future studies could confirm these findings with PSG to determine specific relation to sleep stages and parameters.

It is unclear from this study whether the changes in sleep are precipitants of attacks or are a manifestation of the migraine attack premonitory symptoms. For example, nocturnal polyuria is a common premonitory symptom [45] in which participants wake up often to use the bathroom. Therefore, this could be contributing to interrupted sleep. Nonetheless, changes in sleep detected in this case simply by a smartphone, could be used as a marker of an ensuing migraine attack. This could help migraine patients by having a greater understanding of trigger factors. Moreover, we found that more painful attacks lead to increased sleep duration. This might be explained in that more painful attacks could be accompanied by increased photophobia and users would not wish to use their phone as much during such attacks.

As touched upon previously, we did not have control over whether users napped, and it was not possible to tell in this analysis if naps were included in the sleep duration measures. However, daytime naps were unlikely to have been included in the analysis as the app sets up automatic detection between times which the user can pre-set and would not record outside of these times. Moreover, if naps were included then it is unlikely to be of concern for these findings as we were interested in sleep duration and fragmentation. Daytime naps usually decrease homeostatic sleep pressure evidenced by an increase in SOL [46] and those who are frequent nappers are shown to have reduced SWS during nocturnal sleep but no difference in TST [47]. Thus, suggesting that napping is unlikely to have confounded the results in this study.

Furthermore, these studies were collected over a six-month period from June to December encapsulating both summer and winter months across hemispheres. Recent evidence has suggested seasonal changes in REM sleep, with a reduction in the winter months [48] and a greater preponderance of migraine attacks during the autumn in Europe [49]. Interesting differences in the predictability of migraine attacks using sleep could emerge when comparing seasons and thus future research could explore this, taking into consideration hemispheric differences.

Moreover, another consideration is that although the app collects information covering the ICHD-3 criteria (symptoms, duration, location of pain), we did not verify that each individual user had a migraine diagnosis. Other studies using smartphone headache diaries have reported that only 1/3 of headache attacks met the criteria for migraine [50]. Future studies could verify which users could be diagnosed with migraine according to the criteria. Nevertheless, based on the data, it is reasonable to assume that most of the reported headaches are indeed migraine attacks. Indeed, analysis of the overall symptom reports associated with self-reported migraine “attacks” supports this assumption, with throbbing or pulsating pain being the most frequently reported symptom, closely followed by photophobia. Whilst the current study aids our understanding more generally of sleep and headaches, using these symptoms would allow for more detailed investigations of headache disorders in relation to sleep variables (e.g. migraine with versus without aura). Moreover, we did not analyze any sub-group differences between those with episodic and chronic migraine. This is due to the prevalence of chronic migraine being much lower than episodic migraine in the sample (10.20% vs 89.80%) and thus any differences in sleep or prediction would be difficult to interpret.

We defined attacks based on when the user set the start and end time, with the assumption that the user would most likely report an attack start based on throbbing head pain. This means that if there were two attacks in succession these were counted as two separate attacks. However, it is possible that if a patient took acute medication, the pain would be temporarily relieved, and users reported that the attack stopped. However, the pain could resume, and the user could initiate it as a new attack, when it was in fact the same attack, leading to an overestimate of attack frequency. Although, the user was able to amend the start and end times of their attacks, so this is unlikely to be the case.

Another consideration is that we did not investigate the presence of sleep disorders, psychiatric co-morbidities, or different chronotypes in this sample, yet it is possible that the relationship between sleep and migraine could differ in those with sleep disorders or between chronotypes. Research has shown that migraine patients tend to be extreme chronotypes versus migraine-free individuals, and chronotype influences the number and duration of migraine attacks [51], thus our sample could reflect those with only extreme chronotypes. Furthermore, insomnia commonly manifests as disrupted sleep continuity and insomnia is associated with higher risk of migraine [52], thus the results herein could be influenced by a high prevalence of insomnia. Moreover, there are psychiatric co-morbidities with chronic migraine which could be influencing the results herein [53].

Moreover, the data were collected from the app during the COVID-19 pandemic. Recent reports have suggested that both sleep disturbance and headache are common when suffering from long COVID in response to SARS-COV2 infection [54, 55], thus this may have been a confounding factor. However, given our time frame, number of users and the fact that in most patients COVID-related symptoms are short-lived, this is unlikely to have had a major effect on our data. Future work may want to record SARS-COV2 infections and explore the relationship with headache and sleep disturbance.

Additionally, it is possible that our results were confounded by medication use. Although this information was collected, we had no control over which medications patients were taking and when, and the availability of certain medications could differ across the sample. For example, melatonin for headache prevention is available over the counter in the USA which forms a large proportion of the current sample. This being said, melatonin is thought to affect sleep onset latency but no other aspects of sleep architecture, thus this was unlikely to have affected the results herein [56]. Future studies could investigate the impact of medication on this relationship, assessing whether some medications are more effective at treating those attacks predicted by poor sleep, or assessing whether treatments delivered at certain times may be more beneficial than others. Furthermore, we did not report whether users were taking sleep medication. Chronic migraine patients tend to report using more medication for sleep [57] which could confound the results. Moreover, the only pain measure we analyzed was pain intensity. Other measures of pain could also relate to sleep, such as the physical location of pain, and the presence and location of allodynia. Alternatively, other measures of sleep could also relate to pain e.g. daytime sleepiness.

### Clinical implications

The clinical implications of the current results are that poor sleep could be a predictor of an ensuing migraine attack. Moreover, sleep hygiene should be considered an inherent part of migraine management [58]. As we have shown here, these sleep parameters may be readily detectable by a simple smartphone application meaning minimal input from the patient. This might be aided using sleep diaries and/or wearable sleep monitors. Rather than focusing on the absolute duration of sleep, clinical care and subsequent research should concentrate on ensuring sleep maintenance and reducing awakenings, to reduce the likelihood of attack occurrence. For example, one potential way of fostering sleep continuity is with closed-loop auditory stimulation paradigms. For example, 50 ms bursts of pink noise are played to the up-states of slow oscillations during non-rapid-eye-movement sleep which enhances the depth of slow wave sleep and may ensure sleep continuity [59]. Focusing on reducing pain associated with attacks rather than focusing on total attack prevention, could lessen the impact on subsequent sleep and likelihood of attack occurrence and chronification.

## Conclusions

This study aimed to disentangle the relationship between sleep and migraine. Sleep interruptions and deviation from average hours slept are associated with greater likelihood of a migraine attack occurring, whereas overall sleep duration is not. Conversely, simply experiencing a migraine attack does not predict altered sleep duration but having a more intensely painful attack predicts increased sleep duration. Clinicians should ensure sleep hygiene is intrinsic to migraine management and poor sleep could be considered a biomarker or risk factor for migraine attacks. Longitudinal studies with polysomnography and more detailed investigations of headache disorders classified according to ICHD-III criteria are required to confirm these findings, and methods for improving sleep continuity should be explored as a potential migraine intervention.

### Supplementary Information

**Additional file 1: Supplementary Table 1.** STROBE Statement -checklist of items that should be included in reports of observational studies.

## Data Availability

Data availability: data analysis scripts are available at https://github.com/jackbrookes/healint
